# Engineering organs, hopes and hybridity: considerations on the social potentialities of xenotransplantation

**DOI:** 10.1136/medhum-2024-013061

**Published:** 2024-12-19

**Authors:** Johannes Kögel, Peta S Cook, Nik Brown, Amy Clare, Megan H Glick, Kristofer Hansson, Markus Idvall, Susanne Lundin, Mike Michael, Sofie á Rogvi, Lesley A Sharp

**Affiliations:** 1 Independent, Kottmar, Germany; 2 School of Social Sciences, University of Tasmania, Hobart, Tasmania, Australia; 3 Department of Sociology, University of York, York, UK; 4 Department of Science, Technology and Society (STS), School of Social Sciences and Technology, Technical University of Munich, Munich, Germany; 5 Department of American Studies, Wesleyan University, Middletown, CT, USA; 6 Department of Social Work, Malmo University, Faculty of Health and Society, Malmo, Sweden; 7 Department of Ethnology, History of Religions and Gender Studies, Stockholm University, Stockholm, Sweden; 8 Department of Arts and Cultural Sciences, Lund University, Lund, Sweden; 9 SPSPA, University of Exeter, Exeter, UK; 10 Section for Health Services Research, Department of Public Health, University of Copenhagen, Copenhagen, Denmark; 11 Departments of Anthropology, Barnard College and Sociomedical Sciences, Columbia University, New York, New York, USA

**Keywords:** Anthropology, Medical humanities, Social science, Qualitative Research, Public health

## Abstract

The development of replacing human organs with those from genetically modified pigs holds immense potential for alleviating the shortage of organs necessary for patients in need of transplants. This medical advancement is also accompanied by significant social changes, including the emergence of a bioeconomy, new modes of biotechnology governance, altered human-animal relations and increased public engagement. Some aspects, such as the impact on the transplant allocation system, effects on clinical practice and healthcare provision, global trajectories and most importantly the consequences for patients and their families remain unpredictable. Given that xenotransplantation occurs within a societal context and its success or failure will not be confined to technical feasibility alone, it is essential to engage a social sciences perspective to highlight the social implications and emphasise the importance of social research in accompanying future developments.

## Introduction

 The world has witnessed several efforts in xenotransplantation involving the implantation of genetically engineered organs derived from pigs into human patients. Specifically, these include two heart and two kidney surgeries in the USA and a liver transplant in China. The heart transplantation patients survived 6 and 8 weeks, the kidney patients 2 months. For the liver transplantation patient from China, there is no news available.^1^ Xenotransplantation has also seen initial exemplifications as a subject of policymaking. It marked the first instance in which the precautionary principle was advised in the realm of bioethics (Sobbrio and Jorqui 2014). Additionally, xenotransplantation has prompted public consultation projects in several countries as well as an European Union-wide research project on participatory technology assessment (Griessler *et al* 2012). In social research, xenotransplantation serves as an instructive example to illustrate forms of governance and control such as ‘pre-emptive’ biopolitics and various social relations (including those associated with patienthood, animal ethics, biocapital and expertise).

Xenotransplantation is not a new science and there are documented experiments dating back to at least the 1600s (Deschamps *et al* 2005) with an intensification of sporadic attempts in the USA especially during the mid-to-late 20th century involving non-human primates as source animals and human patients. Most recently, novel scientific insights have emerged with the development of the gene editing system CRISPR-Cas (Clustered Regularly Interspaced Short Palindromic Repeats), a tool that scientists working in the field of xenotransplantation praise for its technoscientific capacities to overcome previous hurdles like porcine endogenous retroviruses alongside a host of other transspecies immunological challenges. In addition to the recent pig-to-human transplantations, experiments with genetically engineered pig organs have been conducted on brain-dead individuals in the USA and in China, attempting to supplement the preclinical non-human primate model (Montgomery *et al* 2024). Yet despite these developments and changes in the scientific and clinical landscape of xenotransplantation, many social and ethical aspects persist.

Considering these developments, we deemed it timely to convene social science and humanities scholars—working in sociology, ethnology, sociocultural and medical anthropology, American studies and science, technology and society studies—dedicated to researching various angles, fields and dimensions of xenotransplantation. Some of us have been working in this field since the 1990s. The conference ‘XenoSocial: Examining the Social Implications of Xenotransplantation’, held in Tutzing, Germany, from 30 November 2023 to 2 December 2023, brought us together to examine the issues of xenotransplantation subjectivities and human–animal relations, public perceptions of xenotransplantation and science-public interaction as well as regulation and governance.

It is not feasible to detail all the results and insights on the social implications of xenotransplantation here. Instead, we would like to highlight the issues that were discussed at the conference before delving into the social issues and questions we believe are necessary for future research endeavours.

## Engineering biological and social technologies

The biomedical and wider ‘sociotechnical imaginary’ (Jasanoff and Kim 2015)—the collectively held visions of (supposedly) desirable futures shaped by the relationship across social, technological, and policy domains—regarding its targets and respective solutions has changed over the last few decades, giving rise to new research strategies and immunitary paradigms (Brown 2019) of which the production of human-animal hybridity emerges as a potentially viable and acceptable technology in saving human lives and alleviating the shortage of human donor organs. Creating interspecies chimaeras by means of inducing human pluripotent stem cells into pig blastocysts or embryos that are eventually able to render individually tailored organs for transplant patients is one of those ideas pursued in experimental research (Casal and Williams 2019; Loike and Kadish 2018).

We observe a development that increasingly operates under the distinct logic of pre-emption which appears to accompany xenotransplantation as an entrepreneurial project. This logic involves pre-emptive medical visions of transplanting organs before they show signs of failure (Cooper 2020; Jagdale *et al* 2019). Importantly, the increasingly commercially driven nature of the xenotransplantation project shows signs of a biopolitics of pre-emption (Carr 2022)—a form of governance that aims to anticipate the future by changing it (through the use of biotechnologies)—guided by a vision directed toward a horizon fraught with potential health or security crises. In this respect, there are some companies that primarily invest in potential future revenues on biocapital—venture biocapital, so to speak—thereby intensifying the pressure to produce xenotransplantation results with less or little regard for non-economic factors (for example, social acceptability, animal ethics or sustainability). On the regulatory side of these processes, we observe the emergence of new forms of regulations such as hybrid regulatory agencies—or ‘institutional hybrids’ (Brown and Michael 2004)—taking shape, which concentrate regulatory powers and reduce diversity in the scientific performances and potentialities of xenotransplantation (Cook *et al* 2011).

## Engineering public opinion

We observe a strong inclination within the xenotransplantation enterprise actively to seek public acceptance as a means to socially legitimise its research and medical interventions. The interface and realm of interactions between science and the public take the form of particular ‘ethno-epistemic assemblages’ (Irwin and Michael 2003; Michael and Brown 2005), increasingly composed of companies advocating for the agenda of xenotransplantation.

Contrary to past expectations based on the deficit model of public understanding of science where public approval of xenotransplantation was presumed once citizens were informed, we have seen varied outcomes from public consultation efforts. In Canada (Einsiedel 2002), Australia (Cook 2011) and the Netherlands (Versteeg and Loeber 2011), moratoriums were endorsed. In New Zealand (Thomas 2007), Switzerland (Griessler 2011) and Germany (Kögel and Marckmann 2020), the outcomes were in favour of the continuation of xenotransplantation research. However, in Switzerland and the Netherlands, the results of the participation formats were not considered by lawmakers even though the process was initiated by authorities (Griessler *et al* 2012), which undermines citizen participation. While the opinions of various ‘publics-in-particular’ (Michael 2009) in these public consultations are valuable, they cannot be regarded as reflective of public acceptance across the entire population, nor should they be dismissed.

## Engineering ‘donors’ and recipients

In terms of the non-human animals involved, the ethical focus is often restricted to comparing the relatively small number of pigs used for research with the much larger numbers of animals farmed for meat production. While using animals as a source of ‘spare parts’ for humans may align with an anthropocentric worldview, it is essential to insist that the frequently made assumption—that using animals to save human lives is acceptable when people also consume them for pleasure—is not uncontested (Koplin 2020). From a biocentric perspective, which underlies animal rights activism, dietary choices such as vegetarianism and veganism, both of which are gaining prominence, particularly in countries where xenotransplantation research is conducted, may influence public perception and acceptance.

It is crucial to note that using animals in xenotransplantation does not equate the life of one pig to the life of one human (Baban *et al* 2023). In the production process of genetically engineered pigs, less than 1% mature into animals (Entwistle *et al* 2022), and on the path to clinical implementation in humans, a range of non-human primates, who have been used as proxies, will have perished during preclinical trials. In addition, for porcine islet cell xenotransplants, 10 adult or more than 90 juvenile pigs would be required for one person (Coe *et al* 2020). Clearly, the number of animals needed for experimental and clinical xenotransplantation is significant.

The acceptability of animal use for human purposes constitutes one aspect of the ‘xenotransplantation paradox’ (Haddow 2021a) which involves the simultaneous emphasis on sameness and difference between humans and animals in xenotransplantation discourse (Brown 1999; Cook 2006, 2013; Hansson 2011; Sharp 2011). The emphasis on similarity across species, as opposed to differences, serves to underscore the feasibility of xenotransplantation. From this framing, physiological differences are not seen as insurmountable barriers. For example, the heart is held to be merely a pump that can function in biological organisms regardless of their species-bound specificity. With the latest focus on gene editing, the legitimising discourses surrounding xenotransplantation have shifted towards a rhetoric of molecularisation (Rose 2001), constructing the argument that it is not only on a physiological level of whole-organ function that pig-to-human xenotransplantation is feasible but also on a molecular one. In this sense, arguments hinge on the notion that scientists can use gene editing technologies to produce porcine organs that exhibit human proteins, which, when transplanted into human bodies, are more compatible with the human immune system on a molecular level due to their transgenic composition and thus improve potential xenotransplantation success.

## Engineering futures and the role of social research

As social science researchers, we acknowledge our role in shaping the social reality of xenotransplantation. With distinct perceptions, interests and normative expectations, we consider it crucial for future developments in xenotransplantation to be accompanied by social research that comprehensively addresses its conditions, implications and consequences.

For the probable event of further xenotransplantation in the future, whether through additional ‘compassionate uses’ or initial clinical trials, it becomes imperative to prioritise the diversity of patient perspectives within the xenotransplantation scenario. This can be achieved particularly through interviews with xenotransplantation recipients as well as with patients who qualify as potential recipients through their diagnosis (such as type 1 diabetes, Parkinson’s disease, cancer) and as (future) research subjects.

### On what we know

Surveys seem to be a wanting instrument for examining a complex topic like xenotransplantation, as knowledge about it cannot be assumed (Cook 2013; Padilla *et al* 2024). It is also difficult to assess the acceptance of xenotransplantation among people who are not in need of one themselves, especially as xenotransplantation tends to evoke strong affective reactions. Consequently, there has been quite a range of differing acceptance rates regarding xenotransplantation, both in public, and patient attitudes studies, with the survey outcomes depending—among other factors—on the information provided or the wording used in the survey (Cook 2013; Hagelin 2004), for example, when juxtaposed with risk perception (Lundin and Idvall 2003). Noteworthy in this regard is that options such as human or artificial organs are preferred among patients and other population groups (Haddow 2021a; Rubaltelli *et al* 2008). This preference has been interpreted as strong affective reactions ([Bibr R62][Bibr R61]). However, qualitative empirical research studies also indicate that xenotransplantation finds acceptance among patients, when no alternative options or treatments exist (Kranenburg *et al* 2005). Accordingly, health matters tend to take priority over ethical considerations, even regarding the use of animals (Kögel *et al* 2021), with the functionality of the transplant important to these patients regardless of the source of the organ (Idvall 2006). The actual risk involved is seen in not receiving a transplant (Lundin and Widner 2000). This is also reflected in paediatric xenotransplantation (Hurst *et al* 2021) where parents of children with congenital heart disease are willing to consider xenotransplantation as a last resort, but are particularly concerned about the stigmatisation of and among children. Nevertheless, social pressure and religious prohibitions are reported to influence patients’ willingness to accept xenotransplants (Şahin Akboğa and Rukiye Hobek 2023). Research with actual xenotransplantation recipients is currently minimal but shows that survival ([Bibr R44][Bibr R45][Bibr R46]) and, among adolescents, autonomy (Terán-Escandón *et al* 2005) take priority over other concerns. In this regard, various justifications for using animals are constructed (Lundin 1999, 2001), leading to entanglements of identity issues and normalisation (Lundin 2002). All these patient groups vary in their diagnosis and do not offer a uniform perspective, hence our calling for future social science research.

Additionally, insights from studies on allotransplantation need consideration, providing potential areas of concern for patients (Fox and Swazey 1974, 1992; Lock 2002; Idvall 2017; Idvall and Lundin 2007; Pearsall *et al* 2002; [Bibr R64][Bibr R65]; [Bibr R67][Bibr R68]; Simmons *et al* 1987). This includes concerns about stigmatisation (as also witnessed with xenotransplantation (Cook 2013)), discomfort with being transplanted, worries regarding one’s subjectivity, identity and embodiment, such as changes in personality or character, considerations of bodily integrity and the symbolic meaning of organs and unrecognised physical, emotional and existential suffering (Sharp 2006). Cultural perspectives and the deeply ingrained symbolic meanings associated with organs persist despite scientific explanations. Thinking within social classifications and normative categories is culturally embedded and not easily dismissed when contrasting it with physiological identities (eg, perceiving the heart solely as a pump regardless of species differences) (Cook and Osbaldiston 2010). Specifically, fleshy organic parts are linked to the identity of their original bearers and the ‘alteration of what you are (in the material bodily sense) does affect who you are (subjectivity) in the case of organ transplantation’ (Haddow 2021b, 161). These concerns are likely to arise, if not more so, in the context of xenotransplantation.

We also expect consequences and implications of xenotransplantation in terms of social inequality. On the one hand, we consider the impact of socially unequal treatments in the current form of allotransplantation on minority groups. Throughout the history of allotransplantation, race has played a complicated role. It has both factored into equations of ‘deserving’ recipients and has troubled those who understand racial identification along the lines of in/authenticity and im/purity. The transplantation of organs across differentially raced bodies has elicited questions about the nature of race itself, from both biological and cultural perspectives (Lederer 2008; Park *et al* 2022). In the case of xenotransplantation, issues concerning race have the potential to expand in complicated ways. While some researchers hail xenotransplants as potentially superior to allotransplants (Hunter 2009), such an understanding fails to take into account social and cultural aversions towards violating the animal–human boundary. It is therefore not clear whether, if given a choice, patients would ever voluntarily opt for a non-human over a human organ. For minoritised groups who have historically been animalised through scientific rhetoric, the stakes of crossing this boundary are even higher (Glick 2018). While the perspectives of minority groups on xenotransplantation have been examined occasionally (eg, Padilla *et al* 2021 assessed acceptance across different racial groups in the USA), the views of minorities across various social categories and in different countries require more systematic research.

Moreover, we have also observed challenges in the patient selection process for ‘compassionate use’ (or ‘expanded access’) candidates for xenotransplantation, particularly in the USA, where requirements for psychosocial support and compliance are biased in terms of class, race and dis/ability. This leaves those patients in a particularly vulnerable position with no alternatives once experimental xenotransplantation is offered (Strand 2023).

On the other hand, the debate on global justice (Rothblatt 2004; Sparrow 2009), especially concerning the unequal distribution of risks, has diminished with some even now endorsing xenotransplantation development in selected countries (Rothblatt 2023). Nevertheless, the unequal distribution of risks is still possible through the outsourcing of xenotransplantation experiments and trials to parts of the world where regulatory requirements may be less stringent or where the potential economic benefits of offering niche treatments to medical (xeno)tourists may be highly appealing (Cook 2013; Cook *et al* 2011).

### On what lies ahead

That being said, we recognise that xenotransplantation may represent an opportunity for many patients to alleviate themselves from feelings of guilt towards donors and their relatives, as well as from the realisation that another human has given their life for them. Xenotransplantation raises expectations (Brown and Michael 2003), including the hope to alleviate the organ shortage and the hope among chronically ill and dying patients for a prolonged life. This may lead them to opt for experimental treatments (a facet of this involves the necessary infringement on what is considered a patient’s autonomy (Strand 2023). One of the challenges that patients and their relatives may face can be seen in the dual construction of the xenotransplanted body (viewed as sacred and profane, subjective and depersonalised, both similar and different from the ‘donor’ species; organs as essential and disposable, simultaneously vitalistic and mechanical). These complexities may pose a potential burden for those affected (potential and actual recipients) necessitating careful consideration and grappling with these intricate aspects.

Many factors remain speculative, such as the potential impact of organ transplant commodification on society and the symbolic value of the organ as a gift. Companies producing genetically engineered pigs for xenotransplantation are already implementing different strategies for their ‘products’. Revivicor, which provided the porcine hearts for the first cardiac xenotransplants, and eGenesis, which respectively provided the first kidney, each implemented 10 or more genetic modifications to the source pigs. Meanwhile, scientists in Germany advocate that fewer genetic edits to the source pigs are a safer and more reproducible approach (Kemter *et al* 2020; Wolf *et al* 2023). These competing approaches have the potential to develop into national market strategies as companies work to develop different models of genetically modified pigs as new biocommodities. This could lead to challenges in decision-making options for xenotransplantation patients, and is likely to pose new regulatory challenges (Cook *et al* 2013).

Additionally, there is uncertainty regarding how the provision of xenotransplants will affect allotransplantation: Will it result in the anticipated surplus of organs or will it lead to a decrease in allotransplants as potential donors perceive a diminished need? This outcome hinges on whether xenotransplants prove to be on par, inferior or superior to allografts. However, the outcome also rests on whether potential allotransplant donors (cadaveric and living) will see human organ donation as necessary given the availability of animal sources. In addition, bereaved families who endure significant pain when agreeing to the organ transplantation of a deceased family member (Ralph *et al* 2014) may decide against it if an alternative becomes available. This would inevitably reduce the availability of organs rather than increase them even if xenotransplantation is viable. Furthermore, it is uncertain whether hierarchies of patient accessibility to allo- and xenografts will arise (eg, as shaped by socioeconomic disparities or insured status).

Operating at the extremes of the possible, xenotransplantation—besides creating human–animal hybrids that evoke various transhuman and posthuman imaginaries—also introduces a new type of social figure: The brain-dead individual who is sustained for the sole purpose of experimental xenotransplant trials. This individual is not merely an ‘organ donor’ but rather a body or corpse ‘donor’, representing a socio-technological-legal construct used as an alternative to non-human primates as an experimental research model. The consequences of this trajectory require careful consideration and further research, including various stakeholders and affected groups involved (Parent *et al* 2023).

Lastly, we anticipate ethical questions related to the role and use of animals, which some may perceive to be resolved to resurface as a matter of public opinion due to the current zeitgeist.

Considering these concerns and recognising that social research often unveils facets and implications that might otherwise remain concealed, we deem it essential for the sake of facilitating better-informed and responsible social and technological developments to accompany these dynamics. This entails conducting research on various aspects, including the patient (and relatives’) perspective (consent, autonomy, selection process, identity and subjectivities), the construction of the animals involved, the forms of regulation, biopolitics and bioeconomy (including the competition between various differently engineered pigs/products) in play on a national and global scale, the knowledge constructed through xenotransplantation, public opinion and the various publics produced through xenotransplantation, the opinions of researchers, clinicians and other stakeholders and the interplay between allotransplantation and xenotransplantation, along with their dynamics and management among other relevant factors.

By emphasising the importance of the social issues and challenges discussed in this article, we hope to raise awareness among medical staff, researchers and policymakers involved in xenotransplantation regarding these matters.

## Footnotes

In addition, information on the causes of death of these xenotransplant recipients is scarce. Official statements point to causes beyond the xenotransplantation.
